# Enhanced dose prediction for head and neck cancer artificial intelligence‐driven radiotherapy based on transfer learning with limited training data

**DOI:** 10.1002/acm2.70012

**Published:** 2025-03-14

**Authors:** Hui‐Ju Wang, Austen Maniscalco, David Sher, Mu‐Han Lin, Steve Jiang, Dan Nguyen

**Affiliations:** ^1^ Medical Artificial Intelligence and Automation Laboratory Department of Radiation Oncology University of Texas Southwestern Medical Center Dallas Texas USA

**Keywords:** adaptive therapy, artificial intelligence, deep learning, dose prediction, head and neck

## Abstract

**Purpose:**

Training deep learning dose prediction models for the latest cutting‐edge radiotherapy techniques, such as AI‐based nodal radiotherapy (AINRT) and Daily Adaptive AI‐based nodal radiotherapy (DA‐AINRT), is challenging due to limited data. This study aims to investigate the impact of transfer learning on the predictive performance of an existing clinical dose prediction model and its potential to enhance emerging radiotherapy approaches for head and neck cancer patients.

**Method:**

We evaluated the impact and benefits of transfer learning by fine‐tuning a Hierarchically Densely Connected U‐net on both AINRT and DA‐AINRT patient datasets, creating Model_AINRT_ (Study 1) and Model_DA‐AINRT_ (Study 2). These models were compared against pretrained and baseline models trained from scratch. In Study 3, both fine‐tuned models were tested using DA‐AINRT patients' final adaptive sessions to assess Model_AINRT_ ’s effectiveness on DA‐AINRT patients, given that the primary difference is planning target volume (PTV) sizes between AINRT and DA‐AINRT.

**Result:**

Studies 1 and 2 revealed that the transfer learning model accurately predicted the mean dose within 0.71% and 0.86% of the prescription dose on the test data. This outperformed the pretrained and baseline models, which showed PTV mean dose prediction errors of 2.29% and 1.1% in Study 1, and 2.38% and 2.86% in Study 2 (*P* < 0.05). Additionally, Study 3 demonstrated significant improvements in PTV dose prediction error with Model_DA‐AINRT_, with a mean dose difference of 0.86% ± 0.73% versus 2.26% ± 1.65% (*P* < 0.05). This emphasizes the importance of training models for specific patient cohorts to achieve optimal outcomes.

**Conclusion:**

Applying transfer learning to dose prediction models significantly improves prediction accuracy for PTV while maintaining similar dose performance in predicting organ‐at‐risk (OAR) dose compared to pretrained and baseline models. This approach enhances dose prediction models for novel radiotherapy methods with limited training data.

## 1 INTRODUCTION

Conventional head and neck (H&N) cancer radiotherapy (RT), typically delivered using techniques like definitive intensity‐modulated radiotherapy (IMRT) or volumetric‐modulated arc therapy (VMAT), generally includes elective neck irradiation. This method aims to comprehensively cover the entire nodal region of the neck, ensuring treatment of both undetectable lymph nodes, and potentially malignant. However, it is associated with significant doses of normal organs‐at‐risk (OAR). An innovative development known as AI‐based nodal radiotherapy (AINRT) aims to directly target suspicious lymph nodes, eliminating the need for whole‐neck treatment.^1^ This approach is further integrated into the Daily Adaptive AI‐based nodal radiotherapy (DA‐AINRT) trial, where adaptive radiation therapy (ART) treatment is dynamically adjusted based on anatomical and geometric changes, resulting in reduced target margins and minimized radiation exposure to healthy tissue.^2^ When integrating these advancements into clinical practice, optimizing treatment planning requires careful consideration of various factors to achieve the best balance between planning target volume (PTV) coverage and sparing OARs. This planning process often involves multiple consultations with the physician, which may delay treatment and affect localized tumor control and patient care.^3^, ^4^ Additionally, the planner's experience and personal preferences can significantly influence treatment plan quality. Researchers have turned to AI‐based dose prediction methods to streamline the planning process and maintain high‐quality standards in radiation treatment plans.

In recent years, the rise of deep learning (DL), particularly convolutional neural networks (CNNs), has significantly advanced image classification and processing in radiation oncology,^5^, ^6^ simplifying tasks like image fusion,^7^, ^8^ tumor segmentation,^9^, ^10^, ^11^ and dose distribution prediction.^12^, ^13^, ^14^, ^15^, ^16^, ^17^, ^18^, ^19^, ^20^ CNNs have been integrated into various architectural frameworks, such as ResNet, U‐Net, and DoseNet for dose distribution prediction across different cancer types, each with its own strengths.^13^, ^14^, ^15^, ^16^, ^17^, ^18^, ^19^, ^20^ However, a critical limitation shared by all these methods is their performance dependence on the size of the training dataset. Obtaining a sufficiently large dataset is crucial for developers aiming to train custom models that achieve high accuracy. Specifically, new treatment techniques like AINRT and DA‐AINRT have limited data availability. While data augmentation and synthetic data generation can help mitigate data availability issues, they cannot entirely resolve the data scarcity issue.

A practical solution is to adopt a transfer learning strategy. This technique leverages knowledge from a source model to transfer common features learned from related domains, ultimately enhancing learning for a target dataset. Transfer learning facilitates model development by allowing adaptation and use of pretrained models, eliminating the need to start from scratch. It has proven effective across various applications, such as image classification and segmentation.^21^, ^22^, ^23^ Furthermore, transfer learning has effectively addressed limited patient data in the dose‐volume histogram (DVH) prediction for rectum and bladder in prostate cancer patients.^24^ Additionally, recognizing that cervical and rectal cancers share a common anatomical region and similar OARs, transfer learning has been successfully employed to enhance dose prediction accuracy and consistency in these cases.^25^

In complex H&N cases—especially characterized by small suspicious lymph nodes within constricted PTVs in AINRT plans, as well as tighter PTV margins and daily variability in DA‐AINRT plans—the capacity of transfer learning to adapt to these intricate planning remains undetermined. This paper presents three studies investigating transfer learning's potential in enhancing prediction model performance under limited data conditions for new treatment techniques. In Study 1, we applied transfer learning to a pretrained definitive H&N dose prediction model for AINRT plans. Study 2 extended this approach to DA‐AINRT plans. To evaluate the effectiveness of these adaptations, we compared the performance of pretrained models initially trained on a larger definitive H&N dataset and baseline models trained from scratch with our proposed transfer learning models, Model_AINRT_ (Study 1) and Model_DA‐AINRT_ (Study 2)_._ Study 3 rigorously investigates dosimetric performance of the transfer learning models, Model_AINRT_ and Model_DA‐AINRT_, in ART settings, utilizing data from the final adaptive sessions of DA‐AINRT patients. While Model_DA‐AINRT_ was specifically tailored for DA‐AINRT patients and thus expected to perform optimally within this cohort, our primary objective was to critically assess the clinical applicability of Model_AINRT_ to DA‐AINRT patients since the only difference in the input is different margin sizes for the PTV. Our investigation in Study 3 aimed to reveal whether these input variations would result in significant dosimetric differences, or if the Model_AINRT_ is an acceptable model for DA‐AINRT patients.

The overall goal of the study is to investigate whether transfer learning can be used to overcome the challenge of training DL‐based models with limited data and assess its potential benefits for novel radiotherapy techniques in the dosimetric aspect of treatment planning.

## 2 MATERIAL AND METHODS

### 2.1 AINRT and DA‐AINRT

**FIGURE 1:**
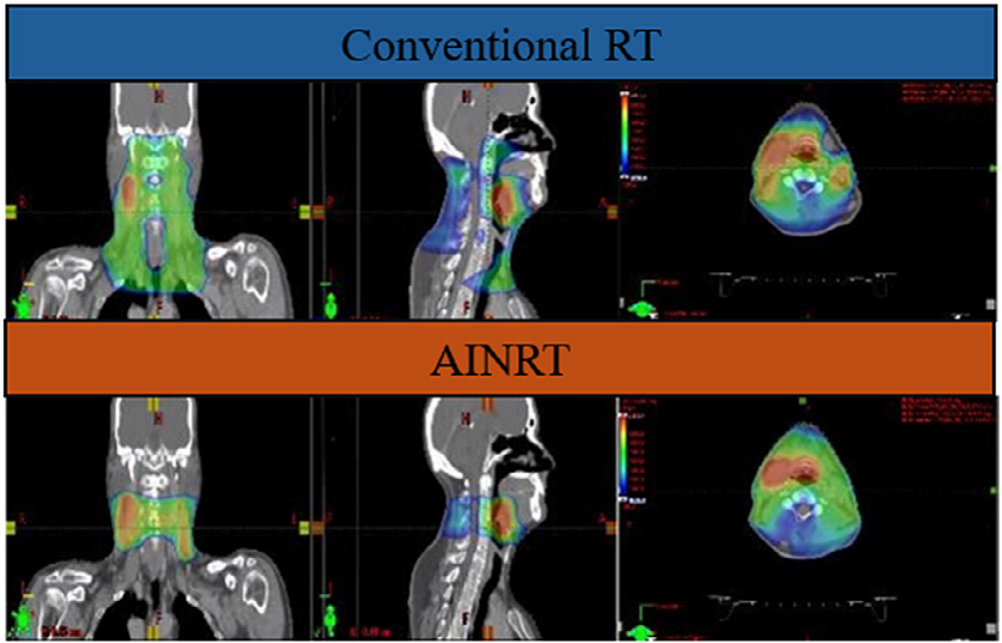
Conventional RT versus AINRT on a dose wash. AINRT, AI‐based nodal radiotherapy.

Conventional radiotherapy approaches for H&N cancer often involve irradiating the entire neck region to account for potentially undetectable small lymph nodes, typically with an 8 to 10 mm margin around the gross tumor volume (GTV), encompassing both the clinical target volume (CTV), and the PTV. Importantly, this approach exposes normal tissues to a significant amount of radiation. However, AINRT,^1^ an innovative approach that utilizes radiologic and radiomic‐based criteria, as well as an AI‐based model to focus on treating suspicious lymph nodes in the neck region, thereby eliminating the need for elective neck treatment of the entire nodal bed (Figure 1). A narrower 5 mm GTV‐to‐PTV margin was used for both the primary and nodal GTVs, as well as the primary CTV, ensuring precise target coverage for AINRT due to the nodes’ small size. Later, this approach led to the integration of DA‐AINRT.^2^ Unlike conventional radiotherapy and AINRT where patients receive a single treatment plan for the entire course, DA‐AINRT adapts to daily changes in patient anatomy, such as weight loss and changes in tumor shape. Daily optimization based on current anatomy allows for substantial margin reduction, employing a smaller 1 mm GTV‐to‐PTV margin (2 mm superior–inferior, 1 mm left–right, and 1 mm anterior–posterior), which further minimizes more radiation exposure to normal tissues.

### 2.2 Transfer learning

In traditional machine learning, an algorithm is trained independently on a large dataset to learn a specific task. This approach, called training from scratch, starts with random initialization of the model's weights and requires substantial data and computational resources to achieve effective performance. Considering the novel treatment approaches and the limited patient data in this study, training a sizeable DL‐based prediction model from scratch with random initialization would be impractical and highly susceptible to overfitting. Instead, we applied transfer learning techniques and utilized the Hierarchically Densely Connected U‐net (HD U‐net) architecture by Nguyen et al.^26^ in 2019 due to its exceptional performance in dose prediction tasks. Compared to standard U‐net, it significantly reduced prediction errors and improved the generalization of image features. Furthermore, it offers a more memory‐efficient alternative, accelerating prediction time.

**FIGURE 2:**
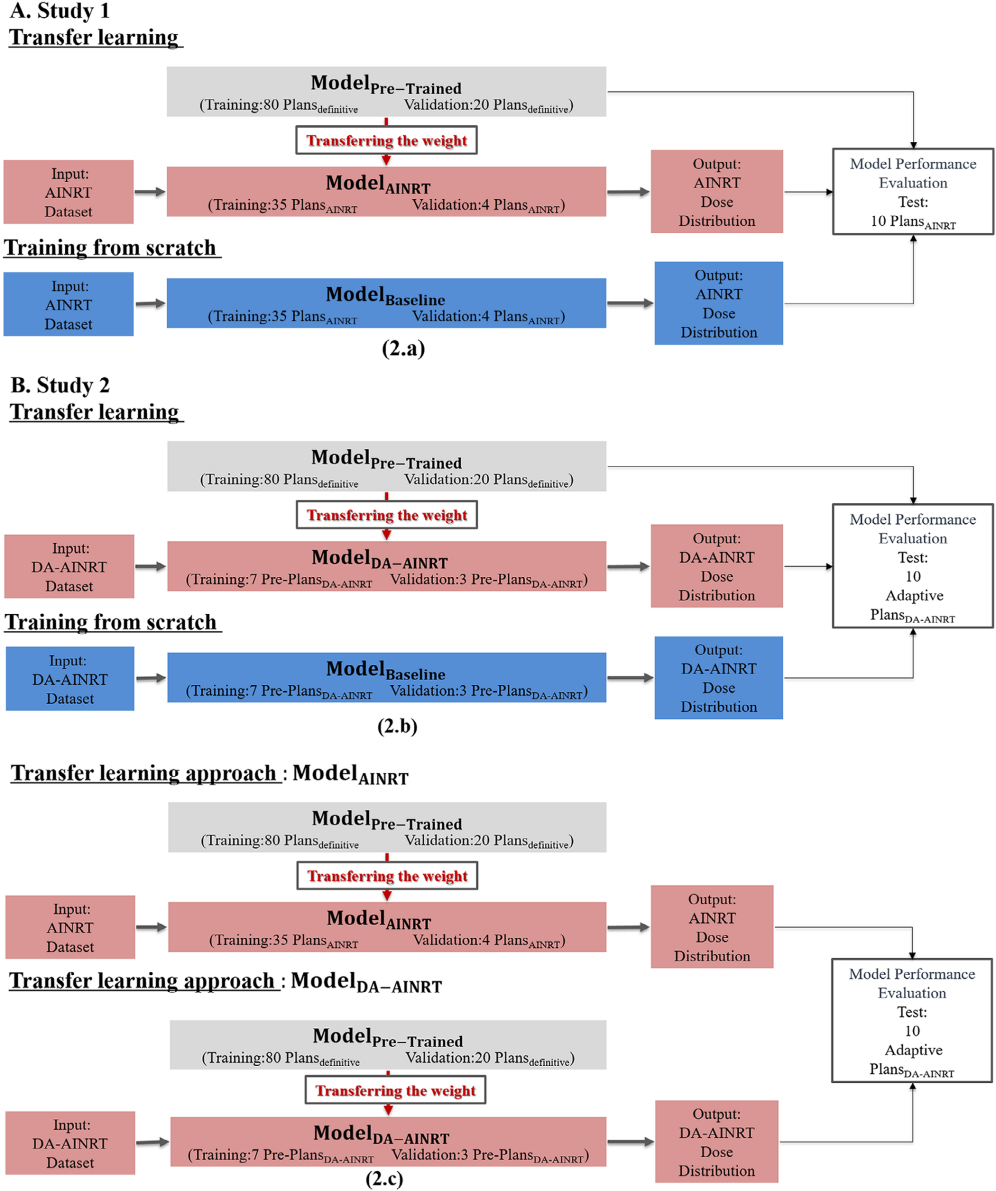
An overview of the transfer learning approach applied across three studies, each with its own dataset split for model training, evaluation, and testing.

Figure 2 provides a comprehensive overview of our transfer learning methodology across three distinct studies, highlighting the progressive refinement in dataset partitioning for model training, evaluation, and testing. This study was approved and authorized by the internal review board for all patient data in this study. In Study 1, we employed the HD U‐net model architecture, training it from random initialization on 95 definitive H&N cancer patients treated with VMAT—80 for training, 5 for validation, and 10 for testing—resulting Model_Pre‐Trained_ (Figure 2a). Because Model_Pre‐Trained_ was developed based on a treatment modality distinct from AINRT, it was subsequently evaluated on a separate set of 10 AINRT cases. This assessment enables an examination of the model's generalization across patient characteristics and treatment approaches that differ from the training data, providing insights into its robustness and adaptability. After validating its performance, we applied transfer learning to adapt Model_Pre‐Trained_ to AINRT treatment data, addressing narrower PTV margins challenge. Specifically, we fine‐tuned the pretrained model on a dataset of 49 AINRT patients, with 35 plans for training, 4 plans for validation, and 10 plans for testing, resulting in Model_AINRT_. In parallel, a Model_Baseline_ was trained from scratch using the same AINRT data and dataset split, allowing for a three‐way comparison between Model_AINRT_, Model_Pre‐Trained_, and Model_Baseline_ to evaluate the impact of transfer learning in addressing data limitations and enhancing accuracy in AINRT predictions. The approved clinical AINRT treatment plans serve as the ground truth, offering a benchmark to evaluate predictive performance across these models.

In Study 2, we utilized the same Model_Pre‐Trained_ from Study 1, initially trained on a cohort of 95 definitive H&N patients, to evaluate its generalizability within the DA‐AINRT treatment approach (Figure 2b). Through transfer learning, we further adapted this model to a dataset of 20 DA‐AINRT patients’ cohorts, including seven pre‐plans for training, three pre‐plans for validation, and 10 last‐session plans as ground truth for testing. This adaptation resulted in Model_DA‐AINRT_, designed specifically to address ART challenges, such as changes in tumor volume and the narrower PTV margins characteristic of DA‐AINRT patients compared to AINRT cases. For a direct performance comparison, Model_Baseline_ was also trained from scratch on the same dataset split, allowing a detailed assessment across Model_Pre‐Trained_, Model_Baseline_, and Model_DA‐AINRT_.

We designed Study 3 to further assess the feasibility of directly applying Model_AINRT_ to predict adaptive session plans and evaluated its performance in this setting since the primary differences between AINRT and DA‐AINRT plans lie in PTV size—affecting radiation precision and effectiveness. This includes examining the accommodation of predicted doses to the PTV and OAR, as well as parameters such as dose conformity and homogeneity. For this analysis, we used the last 10 adaptive session plans from DA‐AINRT patients as the test dataset and ground truth to assess the dosimetric accuracy of both Model_AINRT_ and Model_DA‐AINRT_ in ART settings (Figure 2c).

The patient dataset includes retrospective computed tomography (CT) scans, delineated PTV contours, 42 OARs, and approved doses from radiation oncologists, all obtained using the Eclipse treatment planning system.

### 2.3 Neural network architecture

The HD U‐net was set up as proposed in the paper.^26^ It matched the original HD U‐net architecture with the same number of downsampling and upsampling layers, employing rectified linear unit (ReLU) activation and four max‐pooling layers. Our HD U‐net architecture takes PTV and OAR structures as inputs, with 45 channels. The first three channels represent PTVs, and subsequent 43 channels represent each OAR, segmented into binary masks. Unlike OARs, PTV masks encode prescribed radiation dose. The primary objective of this model is to learn the mapping between OAR binary masks and clinical radiation dose distribution within the body. For model training, a patch‐based approach was used, where 96 × 96 × 64 patches were randomly sampled from patient data to help the model focus on localized areas of dose distribution. Each patch represents a 3D sub‐volume, capturing specific spatial regions containing the PTV and nearby OARs, which allowed the model to learn nuanced dose patterns around these structures. To further enhance model robustness, random data augmentations were applied to each patch, including translations based on the PTV's center of mass, rotations at 0°, 90°, 180°, or 270°, and flips along different axes. Model training was conducted using TensorFlow on an NVIDIA Tesla V100 GPU. Employing the Adam optimizer with a 1 × 10⁻^3^ learning rate, mean squared error (MSE) was the loss function optimized over 100 epochs for each training fold.

This study evaluates dose wash, clinically relevant DVH, and average percent prediction error (APPE) (Equation 1) for key metrics, including OAR mean doses (D_mean_ and D_max_), PTV coverage (D95, D98, and D99), PTV D_mean_ and D_max_, dose homogeneity (Equation 2), and conformity index (CI) (Equation 3). In calculating APPE (Equation 1), *n* represents the total treatment plans, and the summation from *i* = 1 to *n* applies the formula to each individual plan's dose measurement. The ground truth dose is the actual delivered dose, while the predicted dose is the model's output, normalized by the highest prescription dose (70 Gy) to gauge prediction accuracy. These metrics provide a comprehensive evaluation of the model's prediction performance through visual and quantitative analyses. Furthermore, statistical analyses were performed using paired two‐sided *t*‐tests with a significance threshold of 0.05 to compare each model against the transfer learning models. This approach assessed whether observed differences in dose prediction APPE metrics are statistically significant, adding rigor to the evaluation of each model's predictive accuracy and reliability.

(1) $$\begin{array}{ccc} & & \mathrm{APPE}=\frac{1}{n}\sum_{i=1}^{n}\\ & & \;\times \;\left|\frac{\mathrm{Ground}\;\mathrm{Truth}\;{\mathrm{Dose}}_{i}-\mathrm{Prediction}\;{\mathrm{Dose}}_{i}}{\mathrm{Highest}\;\mathrm{Prescription}\;{\mathrm{Dose}}_{i}}\right|*100 \end{array}$$

(2) $$\mathrm{Homogeneity}=\frac{D_{2\%}-D_{98\%}}{D_{50\%}}$$

(3) $$\begin{array}{ccc} & & \mathrm{Conformity}\;\mathrm{Index}\;\left(\mathrm{CI}\right)\\ & & \;=\frac{{\left(\mathrm{Target}\;\mathrm{Volume}\;\cap \;{\mathrm{Isodose}}_{\mathrm{Rx}}\right)}^{2}}{\mathrm{Target}\;\mathrm{Volume}\;\times \;{\mathrm{Isodose}}_{\mathrm{Rx}}} \end{array}$$

## 3 RESULT

### 3.1 Plan dose comparisons

**FIGURE 3:**
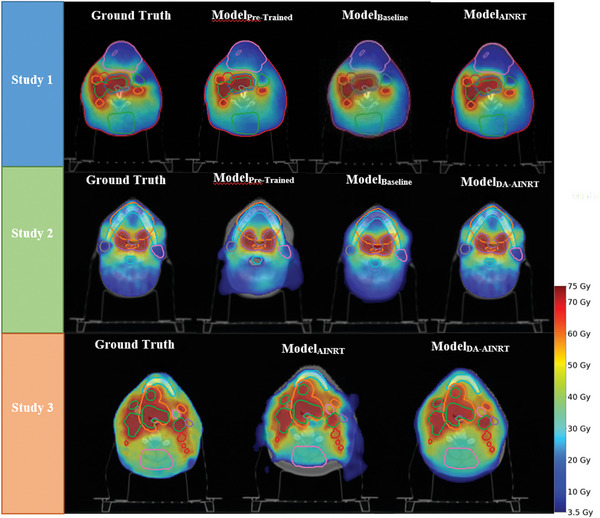
Dose washes for a representative patient from the test pool across three studies. The first and second rows correspond to the first and second studies, showing ground truth followed by predictions from ModelPre‐Trained, ModelBaseline, and transfer learning models. The last row shows ground truth followed by predictions from ModelAINRT and ModelDA‐AINRT. The color bar represents the dose in Gray.

Figure 3 illustrates comparable dose distributions among all three models in both Study 1 and 2. However, a detailed analysis reveals that the transfer learning models outperform the others since the transfer learning models better match the ground truth in dose distribution shapes and dose intensities when compared to Model_Pre‐Trained_ and Model_Baseline_. Shifting the focus to Study 3, Model_AINRT_ displays slight discrepancies in boundary clarity and dose intensity upon visual comparison. Conversely, Model_DA‐AINRT_ achieves improved conformity to the overall dose distribution observed in ART ground truth, characterized by the similar rapid dose fall‐off around PTV coverage and spatial dose distribution characteristics on OARs.

**FIGURE 4:**
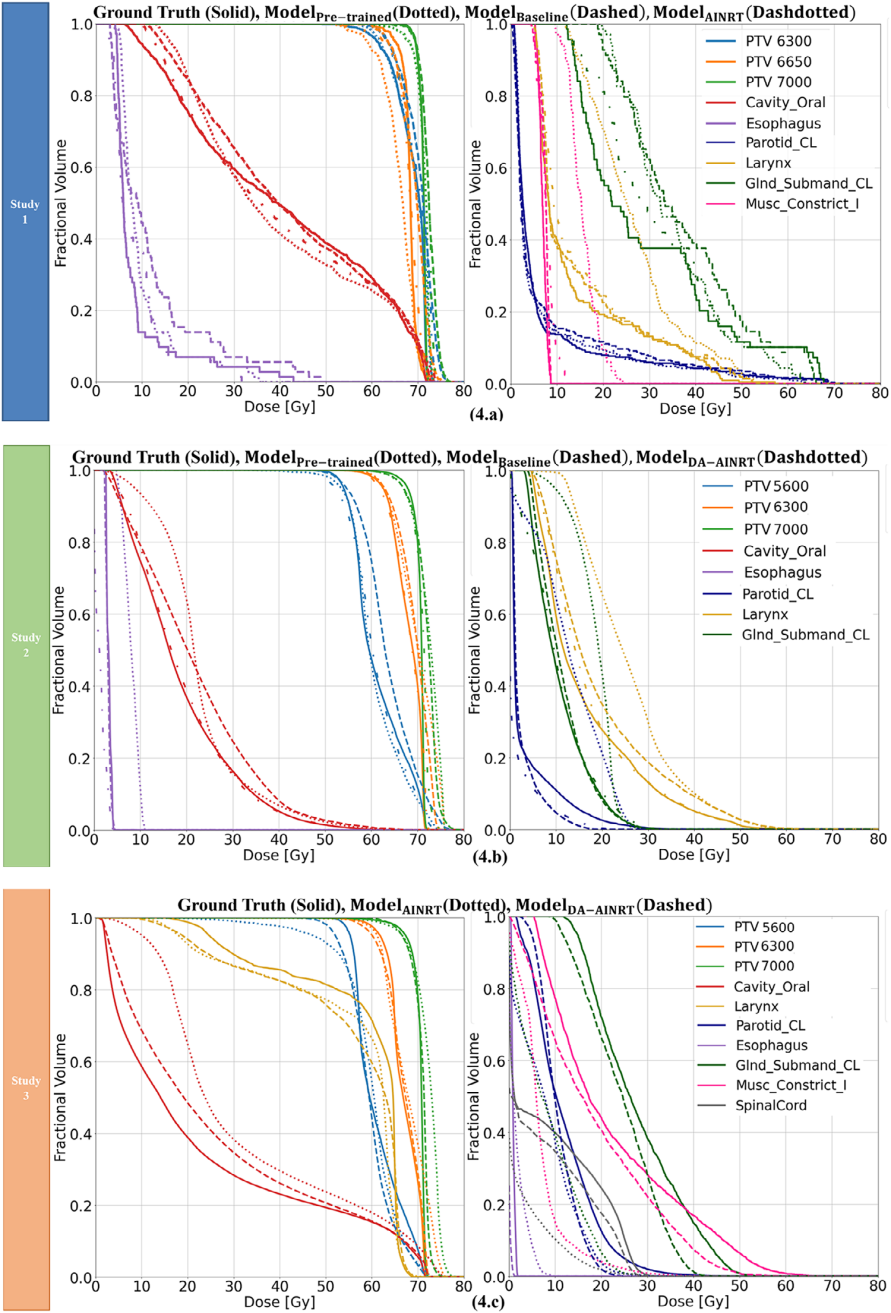
The DVH plot for previous patients from Figure 3 across three studies. (a) Study 1: Solid line—ground truth, dotted line– Model_Pre‐Trained_, dashed line—Model_Baseline_, dash‐dotted line –Model_AINRT_. (b) Study 2: Solid line–ground truth, dotted line–Model_Pre‐Trained_, dashed line–Model_Baseline_, dash‐dotted line–Model_DA‐AINRT_. (c) Study 3: Solid line—ground truth, dotted line—Model_AINRT_, dashed line–Model_DA‐AINRT_. DVH, dose‐volume histogram.

In Figure 4, we evaluated seven high‐impact OARs: the oral cavity, esophagus, contralateral parotid gland (parotid_CL), larynx, contralateral submandibular gland (submandibular gland_CL), inferior constrictor muscle, and spinal cord. The inclusion of these OARs reflects clinical relevance, as documented in the literature,^27^ and acknowledges that not all patients exhibit the same OARs due to variations in tumor location. Here, “CL” denotes “ contralateral,” referring to structures on the opposite side as the primary tumor.

In Study 1, a closer analysis of the PTV regions on the far right of the DVH in Figure 4a, the predictions from Model_Pre‐Trained_ (dotted line) show the greatest deviation from the ground truth, while Model_Baseline_ (dashed line) and Model_AINRT_ (dash‐dotted line) demonstrate closer alignment with the ground truth, though the Model_Baseline_ tends to over‐predict dose in the tail region. Across OARs on the DVH, all models demonstrate similar prediction performance. In Study 2, the predictions from Model_DA‐AINRT_ (dash‐dotted line) align most closely with the ground truth (solid line) across the PTVs on the DVH (Figure 4b), whereas both the Model_Pre‐Trained_ (dotted line) and Model_Baseline_ (dashed line) demonstrate a tendency to over‐predict. For OARs, Model_Pre‐Trained_ consistently over‐predict doses, while Model_Baseline_ and the Model_DA‐AINRT_ exhibit similar DVH profiles, indicating closer alignment with actual dose distributions. Overall, in both Study 1 and Study 2, the transfer learning models show improved prediction accuracy on PTV and maintain comparable performance with the other models on OARs.

In Study 3, Model_DA‐AINRT_ (dashed line) line predicts the PTV 5600 dose closest to the ground truth (solid line), further highlighting its efficacy (Figure 4c). For the other two PTV lines, the dose predictions from Model_AINRT_ (dotted line) and Model_DA‐AINRT_ are comparable to the ground truth, although Model_AINRT_ shows slight over‐prediction in the tail region. Furthermore, the predictions from Model_AINRT_ demonstrate a greater deviation from the ground truth across the OARs, except the larynx.

**FIGURE 5:**
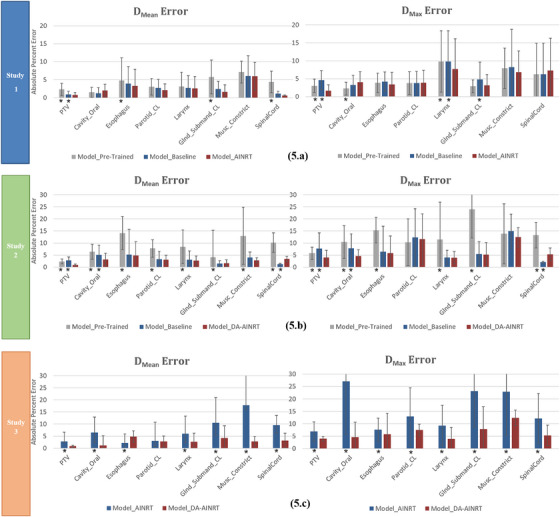
The bar plots of APPE for D_mean_ and D_max_ across three distinct studies. The reported error is presented as a percentage of the prescription dose for the structures of interest. (a) Study 1: Gray bar—Model_Pre‐Trained_, blue bar—Model_Baseline_, red bar—Model_AINRT_. (b) Study 2: Gray bar—Model_Pre‐Trained_, blue bar—Model_Baseline_, red bar—Model_DA‐AINRT_. (c) Study 3: Blue bar—Model_AINRT_, red bar—Model_DA‐AINRT_. Statistically significant differences are marked with an asterisk (*) for *p* < 0.05. APPE, absolute prediction percent error.

After analyzing a representative patient case that illustrates the dose wash and associated DVH, Figure 5 expands the focus to include the entire cohort of test patients. In this broader analysis, the inclusion of error bars provides a visual metric for evaluating each model's prediction accuracy on PTV and OAR relative to the ground truth, using the APPE as defined in Equation (1) to quantify dose deviations across the test cohort.

In Study 1, the error bars in Figure 5a illustrate that, despite being trained on a different dataset, the Model_Pre‐Trained_ demonstrates only a marginal increase in error, indicating that some dose distribution patterns learned during pretraining apply to the target data. Furthermore, the Model_Baseline_ shows a slight reduction in D_mean_ and D_max_ error bars, suggesting a modest improvement when trained on more relevant data. Compared to the other two models, Model_AINRT_ reveals that out of the assessed OARs, six demonstrate reductions in the D_mean_, while four exhibit reductions in the D_max_. Although these improvements in accuracy are not statistically significant, they highlight the potential for further refinement in model predictions. These small enhancements with each model transition underscore that transfer learning can still be effective, even when the differences in dose distribution between models are minimal.

As shown in Figure 5b, the error bars prove that Model_Pre‐Trained_ exhibited significantly higher D_mean_ and D_max_ errors due to its training on data from a different treatment technique. This leads to discrepancies between the pretraining data and the target treatment technique, particularly pronounced since the DA‐AINRT technique has a tighter PTV contour, which substantially affects dose distribution patterns. In contrast, Model_Baseline_, trained from scratch on the target data, presents reduced D_mean_ and D_max_ error bars, indicating an improvement attributed to using more relevant data. Furthermore, Model_DA‐AINRT_ further decreases D_mean_ and D_max_ errors, suggesting that domain adaptation allows the model to fine‐tune its predictions for the target treatment data, achieving the most accurate D_mean_ and D_max_ predictions among the three models. Additionally, the small but notable refinement in accuracy observed in Model_DA‐AINRT_ reflects a meaningful improvement. Both Study 1 and Study 2 demonstrate that the transfer learning models improve PTV prediction accuracy and achieve minor enhancements in OAR dose prediction accuracy, as evidenced by lower error margins.

In Study 3, the error bars in Figure 5c consistently demonstrate significantly lower prediction errors across all OARs and PTVs for both D_mean_ and D_max_ compared to Model_AINRT_. This improvement highlights the impact of domain adaptation in enhancing prediction accuracy. Specifically, the ability to tailor the model to the characteristics of the selected patient cohort appears to play a crucial role in mitigating differences in dosimetry, even though the difference between the characteristics of AINRT and DA‐AINRT is only a smaller PTV size. By training the prediction model on data that closely aligns with the target population, we can better account for variations in anatomy and treatment response, ultimately leading to more reliable dose predictions. These findings underscore the importance of selecting an appropriate patient cohort to optimize the performance of predictive models in radiotherapy.

### 3.2 Statistical analysis

Studies 1 and 2 in Table 1a,b both show variations in D_mean_ and D_max_ errors within the PTV, average PTV dose coverage, and homogeneity between Model_Pre‐Trained_ and Model_Baseline_, making it challenging to conclude which model provides more precise predictions. These differences are sporadic, with instances where one model shows lower errors while the other model performed better in different aspects. However, in Studies 1 and 2, the transfer learning models achieve the lowest prediction errors (highlighted in yellow) for D_mean_ and D_max_ of PTV, average PTV dose coverage, homogeneity (Equation 2), and CI (Equation 3) compared to Model_Pre‐Trained_ and Model_Baseline_. Furthermore, yielded *p*‐values < 0.05, indicating significant differences between transfer learning models and both Model_Pre‐Trained_ and Model_Baseline_. Due to the small test sample size of 10, transfer learning models do not show significant differences in some cases, such as PTV98 and homogeneity against the Model_Baseline_ in Study 1 and PTV99 and homogeneity against Model_Pre‐Trained_ in Study 2. Additionally, while both transfer learning models slightly improve average OAR predictions, these differences are not statistically significant. Overall, the findings highlight substantial improvements in PTV dose prediction, particularly when AI‐detected margins around suspicious lymph nodes are included within the PTV while maintaining comparable OAR dose prediction accuracy.

**TABLE 1:** This table presents the percent prediction errors for D_mean_ and D_max_ in PTV and average OARs, as well as average PTV dose coverage, homogeneity, and CI.

Table 1a				
Study 1				
Model type	PTV D_Max_ error	PTV D_Mean_ error	OARs _Average_ D_Max_ error	OARs _Average_ D_Mean_ error
Model_Pre‐Trained_	3.02 ± 1.84*	2.29 ± 1.70*	5.08 ± 4.43	3.65 ± 3.11
Model_Baseline_	4.60 ± 3.11*	1.10 ± 0.82*	4.67 ± 3.62	3.39 ± 3.31
Model_AINRT_	1.64 ± 1.65	0.71 ± 0.42	4.20 ± 4.65	3.21 ± 3.19

Model type	PTV D95	PTV D98	PTV D99	Homogeneity	CI
Model_Pre‐Trained_	1.55 ± 0.81*	3.75 ± 2.18*	6.40 ± 4.88*	6.2 ± 3.13*	10.61 ± 9.76*
Model_Baseline_	1.90 ± 1.42*	3.46 ± 2.42	5.11 ± 4.15*	6.4 ± 2.09	8.82 ± 8.01*
Model_AINRT_	0.98 ± 2.01	2.65 ± 2.43	4.48 ± 5.06	4.7 ± 2.54	4.90 ± 3.32

Table 1b				
Study 2				
Model type	PTV D_Max_ error	PTV D_Mean_ error	OARs _Average_ D_Max_ error	OARs _Average_ D_Mean_ error
Model_Pre‐Trained_	5.74 ± 2.57*	2.38 ± 1.05*	13.63 ± 9.93*	13.41 ± 7.25*
Model_Baseline_	7.67 ± 3.09*	2.86 ± 1.71*	6.31 ± 3.59	4.47 ± 2.69
Model_DA‐AINRT_	3.98 ± 3.04	0.86 ± 0.73	5.89 ± 2.40	3.32 ± 2.47

**Model type**	**PTV D95**	**PTV D98**	**PTV D99**	**Homogeneity**	**CI**
Model_Pre‐Trained_	2.02 ± 0.94*	2.16 ± 1.03*	2.14 ± 0.98	4.83 ± 2.54*	12.10 ± 13.34*
Model_Baseline_	4.38 ± 1.11*	4.33 ± 1.18*	3.90 ± 1.33*	5.50 ± 2.76*	13.89 ± 11.62*
Model_DA‐AINRT_	1.24 ± 0.60	1.25 ± 0.74	1.34 ± 1.36	3.24 ± 2.13	5.12 ± 4.27

Table 1c				
Study 3				
Model Type	PTV D_Max_ Error	PTV D_Mean_ Error	OARs _Average_ D_Max_ Error	OARs _Average_ D_Mean_ Error
Model_AINRT_	6.87 ± 3.90*	2.26 ± 1.65*	16.77 ± 11.10*	10.41 ± 7.70*
Model_DA‐AINRT_	3.98 ± 3.04	0.86 ± 0.73	5.89 ± 2.40	3.32 ± 2.74

**Model type**	**PTV D95**	**PTV D98**	**PTV D99**	**Homogeneity**	**CI**
Model_AINRT_	3.37 ± 1.57*	3.96 ± 2.42*	4.11 ± 2.08*	6.27 ± 4.23*	12.21 ± 7.90*
Model_DA‐AINRT_	1.24 ± 0.60	1.25 ± 0.74	1.34 ± 1.36	3.24 ± 2.13	5.12 ± 4.27

*Note*: Highlighted colors indicate the model with the lowest error among the three studies. Statistically significant differences are marked with an asterisk (*) for *p* < 0.05.

Abbreviations: AINRT, AI‐based nodal radiotherapy; CI, conformity index, OARs, organ‐at‐risk; PTV, planning target volume.

Lastly, Study 3 in Table 1c reveals that Model_DA‐AINRT_’s predictions in D_mean_ and D_max_ in PTV and average OAR, average PTV dose coverage, homogeneity, and CI on the ART test set significantly differed from those of Model_AINRT_. This highlights the importance of identifying the appropriate patient cohort for training with transfer learning to maximize effectiveness.

## 4 DISCUSSION

In order to assess its application to limited data treatment cohorts like AINRT and DA‐AINRT, our research was fine‐tuned on a pretrained HD U‐Net model,^26^ initially trained on definitive H&N patients. Our comprehensive evaluation framework measures transfer learning's impact and investigates the benefits of transfer learning on dose prediction by considering AI guidance and ART modalities in three scenarios. Our findings in Studies 1 and 2 demonstrate significant enhancements in predictive accuracy for PTV dose predictions with both Model_AINRT_ and Model_DA‐AINRT_ compared to the pretrained and baseline models. These improvements are achieved without increasing OAR prediction error, indicating that the models better target dose distribution to the PTV while maintaining safety for surrounding tissues. In Study 1, we found that the knowledge from Model_Pre‐Trained_ is somewhat transferable to the target data, likely due to similarities in dose distributions between the pretraining and target treatment techniques. This enabled the pretrained model to perform reasonably well despite being trained on a different dataset. The incremental improvements observed with Model_Baseline_ and Model_AINRT_ show that while relevant target‐specific data enhances predictive accuracy, the gains are modest when initial dose distribution differences are minimal. This indicates that transfer learning can be effective when dose distribution patterns are similar, but the benefits of fine‐tuning become more apparent with larger discrepancies, as seen in Study 2. Overall, the use of transfer learning for OAR dose prediction shows only modest improvements, with no significant difference noted. This limited enhancement may be due to the limited sample size of 10 test patients. Additionally, not all patients included all OARs in their contours, and some may lack certain OARs entirely. However, transfer learning significantly improves PTV dose prediction, as accurately targeting a narrow PTV is a primary challenge in both AINRT and DA‐AINRT techniques. This highlights the potential of Model_AINRT_ and Model_DA‐AINRT_ in enhancing target coverage accuracy, reducing irradiation of normal tissues, and protecting critical structures through transfer learning methodologies. In Study 3, using data from the final adaptive sessions of DA‐AINRT patients to test the model, we anticipated strong performance from Model_DA‐AINRT_, specifically designed for this cohort. To compare, we also evaluated Model_AINRT_ on the same patients, where the primary input distinction lay in the smaller PTVs. The results highlighted significant differences in key dosimetric metrics, including PTV dose coverage, homogeneity, CI, and average OAR and PTV D_mean_ and D_max_ prediction errors. These findings emphasize the importance of customizing radiotherapy models to specific patient groups when employing transfer learning to achieve optimal dosimetric outcomes. We can enhance ART's precision and effectiveness by carefully selecting and adapting models based on patient‐specific characteristics.

However, our study has some limitations. First, the excess of transfer learning heavily depends on the quality and quantity of the initial dataset used to develop the pretrained model. Our pretrained model was developed using a relatively small dataset of 80 patients, whereas typical DL algorithms use much larger datasets. Consequently, the transfer learning model might exhibit reduced generalizability and accuracy when applied to new data, as it may not have captured the full variability present in larger datasets. Providing and training the pretrained model with a substantially larger dataset may improve the model's performance. Second, the target dataset (35 plans) is smaller than the source dataset (80 plans), which may cause inconsistent performance between the baseline and pretrained models. This highlights the need for sufficient data in order to make fair model comparisons. Another significant limitation is that the pretrained model was developed without incorporating additional patient‐specific data, such as the physician's preferences. This omission may limit the model's ability to fully account for personalized treatment planning nuances in ART.

Despite these limitations, our study demonstrates that transfer learning has the potential to improve PTV dose prediction while maintaining comparable OAR dose prediction, even in treatment modalities with limited data. It has not only been proven to overcome the challenges of limited data, as seen in other studies^24^, ^25^ that apply transfer learning but also show promise in more complex H&N cases with tighter PTV margins, offering the benefit of enhanced PTV dose prediction without increasing OAR dose prediction error. Future research should focus on expanding datasets and enhancing personalized treatment through patient‐specific modeling and advanced custom architectures for ART. These advancements beyond transfer learning aim to enhance accuracy and efficiency, ultimately improving outcomes for individuals undergoing novel cancer treatment. In addition, although we evaluated the performance of the model using clinical criteria, a reader study to assess the overall acceptability of the model in a clinical setting was not included. In a future study, we also plan to implement the framework in a clinically realistic environment and have a physician evaluate the plan as “acceptable as is,” “requiring nodal/OAR correction,” “requiring planning fine‐tuning,” or “not acceptable.” We expect that such input would provide valuable data on the usability of the model in a clinical setting.

## 5 CONCLUSION

Our proposed transfer learning models, Model_AINRT_ and Model_DA‐AINRT_, consistently predict accurate 3D dose distributions for H&N AINRT and DA‐AINRT plans. These transfer learning models demonstrate enhanced performance compared to pretrained and baseline models. The proposed transfer learning approach addresses the challenges of limited data in complex H&N cases, potentially improving dose prediction accuracy. Additionally, applying transfer learning to train DL‐based models for specific patient cohorts has the potential to significantly enhance the quality and consistency of radiation therapy plans.

## AUTHOR CONTRIBUTIONS

**Hui‐Ju Wang**: Conceptualization; methodology; data curation; software; formal analysis; investigation; writing—original draft software. **Austen Maniscalco**: Data curation and software. **David Sher**: Conceptualization and methodology. **Mu‐Han Lin**: Resource; conceptualization; and methodology. **Steve Jiang**: Supervision; conceptualization; and methodology. **Dan Nguyen**: Supervision; conceptualization; methodology; and writing—reviewing & editing.

## CONFLICT OF INTEREST STATEMENT

The authors declare no conflicts of interest.
